# Utilization of Machine Learning in the Prediction, Diagnosis, Prognosis, and Management of Chronic Myeloid Leukemia

**DOI:** 10.3390/ijms26062535

**Published:** 2025-03-12

**Authors:** Fabio Stagno, Sabina Russo, Giuseppe Murdaca, Giuseppe Mirabile, Maria Eugenia Alvaro, Maria Elisa Nasso, Mohamed Zemzem, Sebastiano Gangemi, Alessandro Allegra

**Affiliations:** 1 Division of Hematology, Department of Human Pathology in Adulthood and Childhood “Gaetano Barresi”, University of Messina, Via Consolare Valeria, 98125 Messina, Italy; stagnof@unime.it (F.S.); sabina.russo@polime.it (S.R.); giuseppe.mirabile@polime.it (G.M.); mariaeugenia.alvaro@polime.it (M.E.A.); mariaelisa.nasso@polime.it (M.E.N.); zemmed2019@gmail.com (M.Z.); aallegra@unime.it (A.A.); 2 Department of Internal Medicine, University of Genova, 16126 Genova, Italy; 3 Allergology and Clinical Immunology, San Bartolomeo Hospital, 19038 Sarzana, Italy; 4 Allergy and Clinical Immunology Unit, Department of Clinical and Experimental Medicine, University of Messina, Via Consolare Valeria, 98125 Messina, Italy; gangemis@unime.it

**Keywords:** machine learning, chronic myeloid leukemia, algorithms, diagnosis, prognosis

## Abstract

Chronic myeloid leukemia is a clonal hematologic disease characterized by the presence of the Philadelphia chromosome and the BCR::ABL1 fusion protein. Integrating different molecular, genetic, clinical, and laboratory data would improve the diagnostic, prognostic, and predictive sensitivity of chronic myeloid leukemia. However, without artificial intelligence support, managing such a vast volume of data would be impossible. Considering the advancements and growth in machine learning throughout the years, several models and algorithms have been proposed for the management of chronic myeloid leukemia. Here, we provide an overview of recent research that used specific algorithms on patients with chronic myeloid leukemia, highlighting the potential benefits of adopting machine learning in therapeutic contexts as well as its drawbacks. Our analysis demonstrated the great potential for advancing precision treatment in CML through the combination of clinical and genetic data, laboratory testing, and machine learning. We can use these powerful research instruments to unravel the molecular and spatial puzzles of CML by overcoming the current obstacles. A new age of patient-centered hematology care will be ushered in by this, opening the door for improved diagnosis accuracy, sophisticated risk assessment, and customized treatment plans.

## 1. Introduction

### General Consideration on Chronic Myeloid Leukemia and Artificial Intelligence

Chronic myeloid leukemia (CML) is a myeloproliferative neoplasm with an annual incidence of 1–2 cases per 100,000 people [1]. The 5-year survival rate for CML is 70%, and an expected 1310 Americans lost their lives to the disease in 2023, according to data collected through 2018 [1]. CML typically comprises three stages. The majority of patients (85–90%) have chronic-phase (CP) disease, which is defined by a high white blood cell count that includes both mature and myeloid precursor cells. This condition typically lasts three to five years prior to the administration of tyrosine kinase inhibitors (TKIs) [2,3]. Approximately 4–5% of patients have accelerated-phase (AP) CML, which is characterized by cytopenias, a rise in blasts, and additional genetic abnormalities. Blast-phase (BP) CML affects 1% to 2% of individuals at presentation [4,5].

The reciprocal translocation t(9;22) (q34.1;q11.2) produces a fusion protein encoding a 210 (kinase domain) kD oncoprotein that increases cellular proliferation and it is the cause of CML [6]. About 95% of CML patients show the resulting BCR::ABL1 oncogene as a diagnostic characteristic. Until the end of the previous century, drug therapy for CML was limited to nonspecific drugs such as interferon-alpha, hydroxyurea, and busulfan [7]. The development of tyrosine kinase inhibitors (TKIs), which effectively disrupted the interaction between the BCR::ABL1 oncoprotein and adenosine triphosphate, significantly altered the therapeutic landscape of CML. Through 20 years of follow-up, the 10-year survival rate increased from 20% to 80–90% thanks to this “targeted” approach, which changed the natural CML course [8].

To confirm the diagnosis and to obtain information for staging systems, peripheral blood smear examination and bone marrow aspiration are recommended for all patients with suspected CML. Moreover, conventional cytogenetics or fluorescence in situ hybridization (FISH) and molecular tests are used to verify the presence of the Philadelphia (Ph) chromosome—the t(9;22)(q34;q11)—or the Ph-related molecular BCR::ABL1 abnormality, to diagnose typical CML [9]. Additional chromosomal alterations (ACAs) can be found using baseline cytogenetic screening [10]. On the molecular side, the classical BCR::ABL1 fusion proteins are as follows: the p210 (generating e13a2 or e14a2 transcripts), p190 oncoprotein (e1a2/a3 transcripts), p230 (e19a2 transcripts) (p230). The prognosis for p190 CML affected patients can be poorer [11,12].

A differential diagnosis with other hematologic malignancies may be required because the diagnosis of CML is not always straightforward. In actuality, the Ph chromosome is present in roughly 25% of adult patients with acute lymphoid leukemia (ALL) and certain people with essential thrombocythemia. Furthermore, the BCR/ABL fusion protein size in approximately 50% of Ph-positive ALL patients is identical to that in Ph-positive CML. The unique morphological, cytogenetic, and immunological traits of various illnesses must be used to differentiate them [13,14].

Clinical hematology is able to benefit from technology transformation [15], as recent developments in artificial intelligence (AI) and machine learning are having a significant impact on medicine [8]. AI technology can be used to prioritize cases, streamline workflow, and diagnose hematological diseases more quickly by using preliminary stratification [16].

In this review, we attempted to identify the potential applications of AI and, above all, machine learning in CML, highlighting both its merits and drawbacks as well as the potential advantages of applying AI in therapeutic scenarios.

Even though our review is narrative rather than systematic, we made an effort to minimize the likelihood of bias in the literature selection process. Research on artificial intelligence, particularly machine learning, in the English language from 2003 to 2024 was examined. We used the terms “chronic myeloid leukemia” or “Ph positive chronic myeloproliferative neoplasm” and “artificial intelligence” or “machine learning”. “Diagnosis”, “prognosis”, and “treatment” were also utilized.

## 2. Use of AI in CML

### 2.1. Diagnosis Through a Morphological Analysis of Peripheral and Bone Marrow Smears

The morphological evaluation of malignant cells in bone marrow (BM) aspirate and peripheral blood smears has been the primary application of AI methods in the context of CML.

In a previous study, the Swedish team introduced the DiffMasterTM Octavia system. It was based on software for image processing and autonomous cell localization. Artificial neural networks were used in the software’s development for blood cell pre-classification. Regardless of whether the material was aberrant or normal, there was a 91% agreement between the test and manual microscopy. Additionally, the DiffMaster had a little higher sensitivity to detect blast cells than manual optical microscopy. Hence, the authors initially showed that a decision support system and a trained morphologist could produce high-quality leukocyte differential count reports and enhance the diagnosis of hematologic malignancies, such as CML [17].

Recently 10,082 patients with probable hematologic neoplasms participated in a trial conducted by Haferlach et al. All peripheral blood smear samples were independently labeled by knowledgeable technicians, and hematologists subsequently examined them. They then used an ImageNet-pretrained Xception model and a convolutional neural network (CNN) model to identify 21 preset classifications of neoplasms, including CML. The model attained 95% concordance with the pathogenic cases and 96% accuracy on the hold-out-set [18].

Another study reported similar findings [19]. The CNN architecture of deep learning for the detection of CML and other leukemia subtypes was evaluated by the authors using data from two public repository databases, the American Society of Hematology (ASH) Image Bank and ALL-IDB. The study showed that the CNN model had an accuracy of 81.74% in multi-class classification of all leukemia subtypes and 88.25% in leukemia versus healthy analysis. Furthermore, as compared to other machine learning techniques, the CNN model performed better [19]. Additionally, another study made use of the same dataset (ALLIDB and ASH), and the authors wanted to explore a framework based on the Internet of Medical Things (IoMT) to better identify the four primary subtypes of leukemia—acute and chronic myeloid leukemias, acute and chronic lymphoid leukemias. The accuracy of Dense CNN (DenseNet-121) and Residual CNN (ResNet-34) was 99.56% and 99.91%, respectively. The suggested IoMT system connected clinical devices to network resources via cloud computing, enabling a rapid and secure leukemia diagnosis [19].

In 2020, a different CNN application for CML diagnosis was also reported. Of the 104 BM smears included in the study, 18 were the outcome of the CML patients. The authors used three distinct CNN frameworks and used transfer learning to increase the model’s prediction accuracy. In the CML subset, the CNN application’s prediction accuracy was 95%, demonstrating the viability of using CNN in conjunction with transfer learning to classify and diagnose leukemic cell morphology. Furthermore, compared to traditional manual optical microscopy, this approach resulted fast, more precise, and more objective [20].

In a study examining megakaryocyte shape, the authors discovered a statistically significant correlation between early molecular response and molecularly undetectable leukemia and loose megakaryocyte cluster forms [21].

A conditional generative adversarial network (cGAN)-based model for the morphological analysis of bone marrow biopsy was reported by another team of researchers. Specifically, the AI model was created to separate myeloid cells from megakaryocytes, and the statistical features of these cells were taken out and contrasted between CML patients and controls. When compared to seven other deep learning-based models, the cGAN performed better in segmentation. Images from 31 healthy participants and 58 CML cases during the clinical validation phase attested to the cGAN-based model’s high accuracy [22]. The number of MKs, the number of myeloid cells, the density of MKs, and the parameters of the MK size (maximum, minimum, mean) were the dimensional statistical features of the multiclass of bone marrow cells that were produced based on the data. The MKs typically seem smaller than their normal counterparts in CML and have a variety of distinctive morphologic features in MPNs. According to the study, the suggested CMLcGAN demonstrated a high diagnostic accuracy in identifying the atypical MKs in CML bone marrow samples, suggesting a potential means of supporting hematopathologists. Another study revealed similar findings [23] (Table 1).

### 2.2. AI’s Application in Immunophenotyping for CML Diagnosis

Since in CML patients, mature neutrophils may exhibit an antigen expression behavior comparable to that of normal neutrophils, immunophenotyping is challenging to apply [24,25]. The potential of utilizing AI to improve the definition of flow cytometric investigations has been discussed briefly in a few works. Cerrato et al. conducted a study and created an image-processing algorithm to diagnose leukemia with different techniques. The algorithm was trained on 1009 pictures of peripheral blood smears and bone marrow aspirates from patients who had been immunophenotypically diagnosed with leukemia [26]. This approach was used in a sample of 341 patients who presented leukemia symptoms, and it was then assessed by a skilled hematologist for external validation. On average, it took 24 h for 75% of people and 48 h for 24% of people to receive an initial leukemia diagnosis. Flow cytometric diagnosis and the ML diagnosis were found to be 95% compatible. Nevertheless, neither the training and testing stages of the ML model nor the ML technique utilized to create the image processing software were discussed in this study [26]. Ni et al. analyzed several cell features and classified the neutrophils as either normal or mature malignant (from CML patients) using support vector machine methods and flow cytometry data [27]. The authors used a support vector machine and a four-color panel to identify CD45, CD65s, CD15, and CD11b in order to distinguish between normal neutrophils and mature neutrophils from patients with CML. They made use of LIBLINEAR, one of the most well-liked SVM packages that can be utilized for linear classification of big sparse datasets with a lot of features and cases. The svm-scale.exe command toolkit from the LIBSVM package was used to rescale nine label-instance data files from mature neutrophils of CML patients and nine files from normal neutrophils. The LIBLINEAR software’s train.exe was used to train the data as the prediction model. The test group’s label-instance data files were rescaled using the training data range that had previously been generated, and the trained prediction model estimated the likelihood that the neutrophils were normal. In order to determine the ideal cut-off probability for differentiating between normal and CML with the best specificity and sensitivity, the data file was processed using receiver operating characteristic analysis. Clinical diagnoses were verified by chromosomal morphology and BCR::ABL1 molecular tests. Using a cut-off value of 51.79% as predicted probability, the model’s overall accuracy was 95.5%, with sensitivity and specificity of 95.8% and 95.3%, respectively. Furthermore, mature neutrophils from patients in the chronic phase and blast crisis of CML did not differ statistically significantly in their predicted probability of being normal mature neutrophils, suggesting that the prediction results are consistent across CML phases [27].

### 2.3. Using AI for Karyotyping in the Diagnosis of CML

Clinicians frequently use the results of karyotyping, a crucial procedure for grouping chromosomes into predefined classifications, to identify genetic disorders and malignancies. However, the diagnostic accuracy and efficiency are decreased by the time-consuming and laborious nature of visual karyotyping that employs microscopic pictures. Even though a lot of work has gone into creating computational techniques for automated karyotyping, none of them can be used without significant human involvement. A study modelled an automatic technique to identify a certain class of chromosomes (class 22) and prescreen for suspected CML to detect abnormal metaphase cells, rather than creating a way to classify all chromosome classes [28]. Three phases made up the scheme: segment individual chromosomes at random, analyze the segmented chromosomes, compute image characteristics to find the candidates, and then use an adaptive matching template to find class 22 chromosomes. To assess the effectiveness of this approach, metaphase cells taken from BM specimens of both positive and negative patients for CML were chosen as the image dataset. In this experiment, 99.3% of cases were correctly classified, with 100% sensitivity and 86.7% specificity [28].

## 3. AI for Improving Adult CML Treatment Response and Disease Progression Prediction Using Biochemical, Biomolecular, and Clinical Data

Enhancing diagnostic, prognostic, and predictive sensitivity for CML would undoubtedly be possible with the capability to incorporate various forms of clinical, genetic, and laboratory data. However, without AI support, managing such a vast volume of data would be impossible. 

ML and deep learning (DL) techniques have been specifically examined in several original studies for the classification and prediction of leukemia. A scoping review assessed the body of research on the subject [29]. Only 12 out of the 176 articles that were first found were selected. The main uses of AI in the management of CML were for diagnosis and classification (75%), then for prognosis and prediction (17%), and therapy (8%).

AI may be divided into three categories for tumor diagnosis: gene profiling-based, clinical parameter-based, and blood smear image-based methods. Support vector machine (SVM), eXtreme gradient boosting (XGBoost), and several neural network techniques, like Artificial Neural Network (ANN), are the most widely used AI models. For instance, MayGAN achieved 99.8% accuracy and high performance in detecting CML, whereas the hybrid convolutional neural network with interactive autodidactic school (HCNN-IAS) reached 100% accuracy and sensitivity in organizing leukemia data kinds [29]. As a result, AI provides novel insights and instruments to improve diagnosis, prognosis, and prediction for chronic myeloid leukemia. Furthermore, by providing sophisticated insights to healthcare professionals, integrated AI solutions enhance clinical results in CML management and optimize patient care.

To estimate 5-year survival in patients with CML, Shanbehzadeh et al. employed eight machine-learning techniques using data from CML patients. These tools included eXtreme gradient boosting, multilayer perceptrons, pattern recognition networks, k-nearest neighborhoods, probabilistic neural networks, SVM, and J-48 [30]. “Full features” and “selected features” were the two datasets into which features were separated. Important characteristics chosen using minimal redundancy and maximal relevance feature selection make up the latter. SCM (kernel = RBF) performed the best on chosen characteristics out of the eight algorithms that were taken into consideration, with accuracy, specificity, and sensitivity of 85.7%, 85%, and 86%, respectively. When full features were considered, the performance decreased in accuracy (69.7%), specificity (69.1%), and sensitivity (71.3%) [30].

The potential of applying AI to assess the likelihood of CML progression has been examined in another study. An application of a commercially available ANN software tool to CML cases exhibiting either blastic phase (BC) or accelerated phase (AP) was previously described [25]. Based on whether the disease had progressed within 18 or 30 months of diagnosis, patients were split into two study groups. The patients were successfully classified by the ANN software based on morphometric, hematologic, and clinical data. This study suggested that early or late disease progression might be demonstrated by commercial software that was not created especially for interpreting CML data [31].

AI techniques can also be used to optimize and customize CML therapy. For example, it can be used to estimate drug response or resistance and choose the best treatment, as patients who are predicted to not achieve MR3.0 within 24 months of first-line imatinib treatment may benefit more from second-generation TKIs like dasatinib or nilotinib.

Banjar and associates created a predictive model on adult CML patients who had imatinib frontline and either attained or failed to achieve MR3.0 [32]. To provide datasets for machine learning, models based on categorization and regression trees (CART), the authors separated the patient cohort into two categories. Predictive assays were taken into consideration together with clinical, molecular, and peripheral blood variables. Over the course of the investigation, six distinct models were created. Every model had a positive predictive value that was more than the other conventional scores that were taken into consideration (73–96% vs. 67%). After considering several factors, model D was determined to be the most effective and subsequently verified externally. Despite the great accuracy of the common prognostic risk scores (Sokal, Euro, and Eutos), the ML-based model had the highest specificity (35%) of any model. It was verified that patients who would not attain MR3.0 for two years were correctly predicted by the ML-based model that was created [32]. 

Additionally, an extreme gradient boosting decision tree-based approach for optimizing the treatment of TKIs in adult CML patients in CP was reported under the name leukemia artificial intelligence program (LEAP) [33]. The study considered 101 factors gathered at diagnosis and included 504 patients in the training/validation cohort and 126 in the test cohort. The following treatment options were taken under consideration: ponatinib, dasatinib, nilotinib, and imatinib. By recommending a treatment that is linked to a higher chance of survival than one that is not recommended by the LEAP model, the study showed how AI may help clinicians [33].

Finally, a study that presented a novel approach capable of forecasting the clinical effectiveness of anti-leukemic medications in patients by transferring traits derived from the gene-expression analysis of various cell lines offered an exceptional, ground-breaking illustration of personalized medicine. The authors analyzed three datasets—two of solid tumor data and one of CML data—and took target drugs into consideration. A total of 28 samples—16 responders and 12 non-responders—treated with imatinib were included among CML patients. Three distinct machine learning techniques—SVM, binary trees, and random forests (RF)—were used in the study to construct predictor-classifiers. While SVM and binary trees’ ideal data transfer parameters enabled a proper separation of clinical responders from non-responders, RF was unsuitable as a data transfer technique in the dataset under consideration [34]. Similarly, in the context of the ENEST clinical trial, a sub-analysis was conducted using an ML method. In this instance, important microRNAs with differential expressions were found to be predictive biomarkers of nilotinib response using an ML-driven approach. Data from 58 patients both before and after nilotinib treatment were considered. Based on RF and Bayesian ML algorithms in conjunction with a survival statistical analysis, the study validated AI’s potential to enable CML treatment optimization by separating responders from non-responders [35] (Table 2).

## 4. AI-Based Assessment of Drug Resistance in CML Patients

Even though TKI therapy works for the majority of CML patients, resistance may develop. Drug efficacy in patients with CML could be improved by using more sensitive techniques, such as AI, mathematical modeling, and computational prediction methods, which could uncover the underlying mechanisms of drug resistance and make it easier to create more efficient treatment plans [36].

The identification of mutations that decrease the affinity of type I and type II inhibitors led to the development of SUSPECT-ABL, a ground-breaking web-based diagnostic tool for predicting resistance dynamics. It was possible to identify and compute the differences in ligand affinities brought by resistance mutations in ABL1. Through in silico saturation mutagenesis, the technique has discovered possibly novel resistance mutations, opening the door for in vivo experimental validation. The suggested strategy is a valuable tool for improving precision medicine efforts and for developing next-generation inhibitors that are less prone to develop resistance. The tool has been made publicly available online by the authors, allowing the scientific community to assess it [37]. In an analogous way, Jie Su et al. [38] used AI to create novel therapeutic compounds to overcome mutations linked to T315I resistance and verified outcomes using in vitro cultures (Table 2).

They specifically evaluated autophagy, apoptosis, cycle arrest, and suppression of BCR-ABL1 phosphorylation.

### AI and Treatment Side Effects in Individuals with CML

An indirect use of AI was demonstrated to detect TKI side effects. Adverse events, including those underreported or preclinical, were predicted using a unique text-mining approach. The authors proposed a novel cross-domain text mining method that used hub node network analysis, link prediction, and a knowledge graph to predict new relationships. Tyrosine kinase-related probable adverse events (AEs) that are less well-known can be found using novel cross-domain text mining across all 30+ million biomedical publications in PubMed. Using bag-of-words cluster analysis, adverse events can be linked to TKI drug classes. Thus, new findings are made available by cross-domain text mining through natural language processing (NLP) and machine learning (ML) [39].

A virtual laboratory called ProTox-II is used to predict the toxicity of small compounds. It combines molecular correlation, pharmacophores, AIGT propensities, and machine-learning models to predict a variety of toxicity endpoints, including toxicity targets, hepatotoxicity, cytotoxicity, carcinogenicity, mutagenicity, immunotoxicity, adverse outcomes pathways (Tox21), and acute toxicity. The predictions are supported by data from in vitro tests as well as in vivo examples.

An allosteric inhibitor called asciminib binds to a myristoyl region of the BCR::ABL1 protein. Asciminib targets both normal and modified BCR::ABL1, including the intermediate T315I mutant [40]. The drug works by locking the BCR::ABL1 into an inactive conformation, which sets it apart from all other ABL kinase inhibitors. Despite having strong selectivity for only ABL1 and ABL2 kinases, it shows minimal action against unmutated BCR::ABL1 and all clinically detected ATP-site mutations, including T315I. This is because the myristoyl pocket has a special shape [41]. Serious hematological abnormalities and/or gastrointestinal side effects are among the health risks associated with an overdose, which are likely to align with the adverse effect profile of asciminib.

The goal of the study was to use the natural vitamin E molecule gamma-tocotrienol as a BCR::ABL1 inhibitor to overcome the toxicity that currently exists in the medications that are currently supplied for (Ph+)leukemia. A toxicity comparison study with asciminib was conducted after the de novo drug design of tocotrienol using an AI DL system [42], and three successful de novo therapeutic compounds for the BCR::ABL1 fusion protein were created using gamma-tocotrienol in an AI drug design system. Out of the three, artificial intelligence gamma-tocotrienol’s (AIGT) drug-likeliness investigation resulted in its designation as a potential target. In addition to being more effective overall, the toxicity assessment study also shows that AIGT is hepatoprotective [42] (Table 2).

## 5. AI-Powered Drug Discovery for CML Patients

AI is a highly effective tool for drug discovery because it can reveal the latent but causal links between the biological and chemical sides [43,44]. A targeted chemical library of compounds can be readily adjusted by setting suitable similarity cut-offs and a number of physicochemical parameters. Retrospective molecular docking simulations can help in silico trials pick drugs for experimental testing in a logical manner [45]. Understanding the importance of allosteric regulatory sites and, thus, the logical design of novel, promising allosteric inhibitors can be greatly aided by in silico techniques. The most widely used methods in this regard are based on ligand- and structure-based techniques. When a target’s homology models or crystallographic solution structures are available, the former is used. The latter is used when structural data information is lacking, making structural similarity to known active compounds crucial for conducting investigations such as pharmacophore modeling and quantitative structure–activity relationships [46,47]. 

Several tools were used. By evaluating the WADDAICA online server, the innovative method of revolutionary AI for drug creation using deep learning algorithms was put into practice.

Because it makes it possible to predict virtually how small chemicals would interact with proteins, such as receptors or enzymes, docking is a useful screening technique. AlphaFold is an artificial intelligence program created by DeepMind that uses a deep learning model to predict the three-dimensional structures of proteins [48]. Moreover, DeepSite is a protein-binding pocket modulator based on deep neural networks.

By offering a thorough test set based on more than 7000 proteins from the scPDB database, a machine learning algorithm that uses DCNNs to predict ligand-binding sites in proteins shows that consumers can capture binding site characteristic features given enough training data [49].

The BCR::ABL1 fusion protein’s PDB file was uploaded to the NVIDIA GPU-equipped server via a WebGL graphical user interface to identify and locate pockets. The AIGT compound was studied using the Molinspiration tool, which helps forecast the likelihood that target molecules will become pharmaceutical medications [50]. This method was used to estimate the bioactivity scores of the most significant pharmaceutical targets and to compute key molecular attributes (e.g., logP, polar surface area, number of hydrogen bond donors and acceptors, etc.). Numerous free web-server programs make extensive use of other computer-aided techniques. The AllositePro version 2.10 website provides an example, which is based on a technique for allosteric site prediction utilizing an SVM based on perturbation analysis in conjunction with topological and physiochemical pocket data [51]. As an alternative, CavityPlus finds potential binding sites on the surface of a certain protein structure and ranks them based on ligandability and druggability criteria [52].

The Kinase Atlas server web application, a curated library of untapped allosteric sites, is another effective implementation for allosteric drug development [53]. The collection is based on 4910 PDB structures of 376 diverse kinds of kinases that have been crystallized. FTMap is a method that uses small organic molecules as probes to identify the so-called binding hot spots [54].

Random forest, support vector machine, and deep neural network techniques have been used in Miljković’s work to create precise and reliable models [55]. A vast database of chemicals experimenting with various binding mechanisms is used to train the models. Allosteric and non-allosteric kinase inhibitors with comparable but different modes of action can be distinguished by the global and balanced models that are produced. These findings are highly appealing since they open a new potential off-patent chemical arena by allowing the exploration of novel scaffolds.

Additionally, two in silico approaches were used to create novel possible small-molecule allosteric TKIs on one side and study the Abl allosteric binding pocket on the other. To clarify the most typical allosteric residues and their energetic contribution, the first analysis specifically used a structure-based approach that was centered on the myristoyl binding site. The second study made use of an internal automated generative ML algorithm that may create a library of novel, potentially selective TKIs with easily adjustable user-specified features [56].

Melge and colleagues validated two distinct ML-supervised models in vitro using CML cell lines. They used AI to create a novel medication that combines two distinct compounds, one of which (ponatinib) targets BCR::ABL1. ML-supervised models created a variety of chemical compounds, but the most promising one showed activity in inhibiting cell line growth in both TKI-sensitive and TKI-resistant cells [57].

Moreover, the utilization of natural compounds to treat CML represents a wide range of AI applications. It has been demonstrated that the secondary metabolite withaferin-A (Wi-A), which is isolated from ashwagandha (*Withania somnifera*), has anticancer properties. The authors found ABL to be a promising candidate after doing an inverse virtual screening to examine its capacity to bind to the catalytic site of protein kinases. The impacts on constitutively active BCR::ABL1 oncogenic signaling that results in unchecked proliferation and apoptosis inhibition in CML were examined using molecular docking and molecular dynamics simulations. Wi-A’s Inverse Virtual Screening as a Protein Kinase Inhibitor Using the xglide.py script of the Schrodinger suite 2018-2, which includes the standard precision method of Glyde, Wi-A was screened for 851 kinase protein–ligand structures that were retrieved from the scPDB database [58,59].

The authors discovered interactions between Wi-A and Withanone (Wi-N), a closely similar withanolide, at the ABL’s catalytic and allosteric regions. When comparing Wi-A to the clinically utilized medications Imatinib and Asciminib, the computed binding energies were higher at the catalytic and allosteric sites, respectively. At the allosteric site, Wi-N’s binding energy was lower than Asciminib’s. When exposed to ligand contact, the conformational changes and interaction were found to be comparable to those of the medications Asciminib and Imatinib [60]. According to the results, Wi-A and Wi-N could be used as natural medications to treat CML. Although much more in vitro and in vivo research is necessary, the inhibition of ABL is proposed as one of the contributing causes to the anti-cancer effect of Wi-A and Wi-N (Table 3).

### Applying AI to the Study of CML’s Molecular Space

Even more pertinent than previously mentioned is the application of AI to the investigation of novel medicinal compounds in CML patients. Due mostly to extensive screening efforts, a wealth of information about how small compounds affect biological systems is growing. We should make significant progress in understanding biological chemistry and in identifying prospective therapeutic chemicals and targets by analyzing such datasets using the kinds of computational methods that the bioinformatics community pioneered [61].

In fact, a crucial factor in understanding the nature of living systems is that biological molecules do not act in isolation in the dilute solutions familiar to most chemists. Instead, they are packed together to an extraordinary degree within cells [62,63].

It is therefore necessary to characterize the various chemical substances present in a microenvironment and evaluate their relationships. Chemicals can be characterized by a wide range of ‘descriptors’, such as their lipophilicity, molecular mass, and topological features. ‘Chemical space’ is a term often used in place of ‘multi-dimensional descriptor space’: it is a region defined by a particular choice of descriptors and the limits placed on them. According to this realization, chemical space is the entire descriptor space that contains all of the tiny carbon-based molecules that are theoretically possible to produce [64].

Although a coordinate-based model of molecular space is commonly used and offers an attractive representation, it has some drawbacks, including high dimensionality. The use of dimensionality reduction techniques is obviously necessary for the display of the high-dimensional chemical space. Principal component analysis, self-organizing maps, stochastic proximity embedding, t-distributed stochastic neighbor embedding, and generative topographic mapping (GTM) are the most often used techniques [65].

A different approach is to compute a coordinate-free space representation using a molecular similarity, in which a node stands for a molecule and an edge stores the similarity between the two molecules it connects [66,67]. By first converting each molecule into its pharmacophore graph, a study suggested visualizing and analyzing a chemical space devoted to BCR-ABL tyrosine kinase inhibitors at the pharmacophore level. The authors of a study presented the pharmacophore network, in which the parent–child relationships between the pharmacophores are the edges and the topological pharmacophores are the nodes [68]. Using the graph edit distance (GED), authors in a previous study spatialized a selection of topological pharmacophores and grouped them into clusters where the elements share important structural characteristics and activity [69].

Numerous drug discovery projects have emerged as a result of imatinib resistance, which has helped chemical databases store thousands of data points. Over 124,000 patterns with at least 10 molecules supporting them and displaying four to seven pharmacophoric properties have been produced by the dataset mining process. In total, 99% of the molecules in the dataset are covered by these patterns. Despite the long-standing benefits of pharmacophore triplets, the authors of this study did not take order three patterns into account because of the size of BCR-ABL inhibitors. Here, the excessive simplicity of O3 patterns makes it difficult to distinguish between big molecules with different general structures but a few essential characteristics in common, limiting their capacity to adequately represent the intricacies of molecular interactions during drug–target binding. The procedure known as maximal marginal relevance feature selection (MMRFS) was used to choose a representative subset of patterns. To prevent selection biases and guarantee thorough coverage of the molecular dataset, the subset was computed before evaluating the patterns’ association with activity. Ninety-eight percent of the sample was covered by the 298 patterns that were identified using MMRFS [68].

This work demonstrated a novel approach that does not require the selection of training sets in advance for autonomously calculating topological pharmacophore hypotheses from large molecular datasets. Before evaluating pharmacophore quality, important changes to the representative pharmacophore hypothesis selection stage guarantee objective subset determination. Additionally, by comparing the extensions of different pharmacophores pairwise, the authors assessed the structural proximities between them. The improvement of node labeling on the pharmacophore space by classifying covered compounds into four activity classes rather than a binary model was one noteworthy accomplishment. By improving the color scheme used in the pharmacophore space, this improvement makes specific areas of interest analysis and visual inspection easier [68].

The knowledge gained from these investigations can be applied to rule-based systems in the future to forecast biological activity thanks to the use of AI.

## 6. Prospects for the Application of AI in CML in the Future

The combined use of AI with other sophisticated investigation methodologies may improve the care of CML patients. Raman spectroscopy, for example, is a non-invasive laser-based vibrational spectroscopy technique that yields a spectral output that can be examined to clarify the sample’s biological makeup [70]. Raman spectroscopy can be used to identify changes in cancer cells that occur in the amounts and/or conformation of proteins, lipids, carbohydrates, and nucleic acids since it gives a fingerprint of molecules [71,72,73,74]. Furthermore, because it is a non-invasive approach, it enables exogenous label-free data gathering at the single-cell level in vitro. Moreover, Raman scattering by molecules adsorbed on rough metal surfaces or by nanostructures like plasmonic-magnetic silica nanotubes is amplified by surface-enhanced Raman spectroscopy, also known as surface-enhanced Raman scattering (SERS) [75,76].

In a study, an ordered array-assisted microfluidic chip and ML analysis were combined to create a SERS-based platform for leukemia phenotyping [77]. Clinical samples’ SERS spectra were gathered and prepared beforehand. Baseline deduction, spectrum smoothing, feature peak fitting, and peak intensity extraction are all part of the pre-processing. With normalized data and automated kernel scaling, the training model was configured as a linear SVM model. To validate the model, a cross-validation method was used. The SVM model improved general usability and expedited the analytical procedure. In clinical blood samples, the model showed an accuracy of up to 98.6%, suggesting its potential for use in leukemia diagnosis. But there are also two issues with the current platform that need to be fixed. First, not all subtypes can be covered by the available library of SERS probes. Second, operators are required since the automation potential of microfluidics has not yet been completely utilized. It is relevant that this technology will reduce the time needed for clinical leukemia typing, which often takes days, to less than one hour with automated microfluidics and a full library of probes [77].

Furthermore, even though many of the most well-known AI applications are based on ML techniques, there are other AI fields that are gaining prominence and attention. Formal ontologies, for instance, are among the most representative. In the subject of automated reasoning, ontologies are especially relevant because the goal is for an agent to be able to make logical deductions based on general principles and known facts. Despite not being as popular as ML-based methods just yet, this class of approaches has a lot of potential advantages: ontologies can store enormous amounts of knowledge, can be verified by human experts, which supports their explainability and reliability, and can be expanded if added information becomes available. Additionally, they can be combined with ML techniques to create synergy [78,79,80]. 

## 7. Conclusions

**Figure 1:**
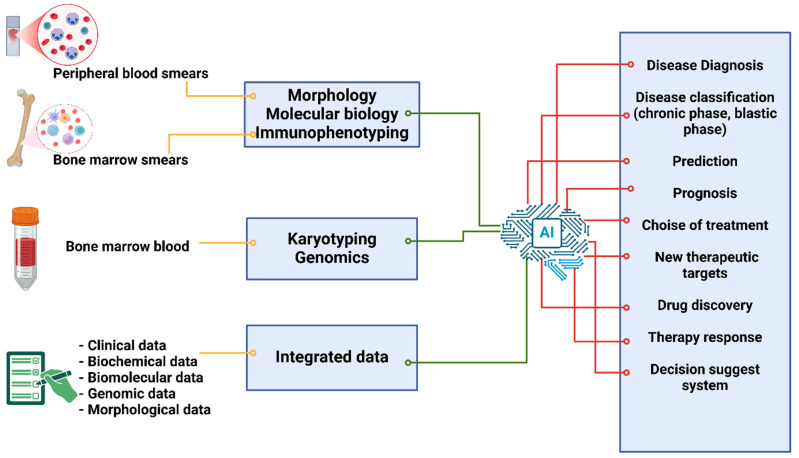
Possible employes of artificial intelligence in the prediction, diagnosis, prognosis, and management of chronic myeloid leukemia.

Through the creation of clinical systems based on guidelines and techniques in data processing and clinical image analysis, AI has changed the diagnosis, treatment, and prognosis of several hematological diseases [81,82] (Figure 1).

While some systems automate the classification and diagnosis of CML using medical pictures, others allow for the early detection of CML based on clinical and laboratory data. Early identification, timely treatment, and better clinical outcomes are possible by these developments. AI provides deep, useful, and non-invasive analytical capabilities in complicated and uncertain settings, including forecasting cancer outcomes and survival, in contrast to conventional statistical and experimental prediction methods [83,84]. 

However, it is crucial to consider the drawbacks of using AI as well. The full and quick adoption of AI in the medical area is hampered by a number of issues, and the extensive use of ML is limited. Issues including inadequate sample numbers and subpar study design must be resolved, and data mutability is another issue with the use of such systems in hematology. Moreover, overfitting, a prevalent issue in machine learning models because of their high degree of data distribution flexibility, presents a distinct challenge. An excellent fit on the training set but subpar performance on the test set may arise from this. Moreover, choosing the right variables is essential when creating a model since adding superfluous or irrelevant variables can degrade its functionality. Finally, algorithms must be developed specifically to enhance clinical workflow in order for DL to be used in clinical practice [85].

Furthermore, one of the most difficult problems in image analysis is reproducibility. Because the cognitive patterns of an AI agent can be obscure, particularly if it is built on an ANN-family algorithm, it can be challenging to determine the involvement of the technology in image acquisition and reconstruction. Results may be impacted by some artifacts that may vary depending on the hardware quality, the set and version of reconstruction methods used, and certain ambient characteristics that may not be reproducible elsewhere. Furthermore, there are serious ethical concerns with AI’s development. The relationship between the decision maker (DM) and the decision support system (DSS) is one aspect of the issue, in addition to privacy concerns (the requirement to retain vast amounts of personal data). Due to cultural and legal ramifications, the DM has historically been the clinician, and the software is a DSS. The DM then decides supported by his knowledge (science) and feelings (art) following consultation with the DSS. The AI decision-making process is obviously entirely different.

Moreover, the “black box” issue, characterized by the opacity and complexity of machine learning model internals, presents a considerable hurdle in medical utilization. It is crucial to develop explainable AI solutions because a lack of transparency may impede patients’ and clinicians’ trust and adoption. Between the demand for intelligible, interpretable predictions and the high performance of contemporary machine learning models, explainable AI seeks to close the gap. The creation of tools like SHAP, LIME, and Influence Functions supports the validity and dependability of the models while also providing transparency [86].

Lastly, because AI systems must be trained or optimized on vast volumes of data, the systems that are produced are often biased in favor of the population from which the data were gathered. The majority of this comes from wealthy Western nations that currently have the resources and infrastructure to allow the establishment of data pipelines and ecosystems for data sharing. Hence, underrepresented populations may not receive the best possible care.

AI is currently capable of delivering exceptional results, especially when it comes to data analysis. Beyond the bounds of human cognitive patterns, it can study relationships between vast collections of covariates or utilize imperfect knowledge to develop precise diagnostic and prognostic predictive models. Along with preventing or treating adverse effects, this also opens up new therapeutic possibilities. With the potential to identify new compounds more quickly, shorten trial durations, and significantly lower costs, the field of new drug exploration in particular seems to be the most promising. 

But there are a number of aspects that need to be considered. As an example, ML is more empirical than classical analysis and is focused on testing outcomes a posteriori. This necessitates a more critical interpretation of the findings. In addition, many of the steps that span from data gathering to model performance evaluation lack standards. 

Additionally, results are still pending external validation with samples drawn from groups that differ from those studied in the original research. Finally, data from a single site or data acquired differently than in clinical practice were used in certain research, which had tiny sample sizes.

However, although the use of AI to treat hematologic malignancies is still a ways off, it is undoubtedly worth considering and researching. It is important to remember that every hematologic disease has unique characteristics that affect the purposes and usefulness of AI applications. Similar to what has already been established in other hematological diseases like acute myeloid leukemia, AI has been demonstrated to be more capable than humans when it comes to analyzing the cytomorphology of bone marrow samples and peripheral blood smears. In CML patients AI may assist researchers in finding novel medications and modifying them to ensure less toxicity, much as what has been shown in other disorders. However, therapy discontinuation is a new area where AI may be crucial for CML patients [87]. After stopping TKI treatment, between 40 and 60 percent of patients who achieve a stable deep molecular response will continue to be in remission; this is known as treatment-free remission (TFR) [88]. After stopping treatment, however, the majority of patients relapse, necessitating the resumption of TKI therapy [89]. AI-powered multiparametric analysis could more reliably identify patients who are ready to stop treatment.

In any case, it is anticipated that AI-driven multimodal data integration and the creation of increasingly intelligent operational platforms would both make it easier to investigate the disease and offer new tools and approaches for better management of CML.

## Figures and Tables

**Table 1:** Algorithms for diagnosis through blood smear images.

Authors—Year	AI	Algorithm	Ref.
Ahmed et al.—2019	CNN model was trained with 25 epochs and 32 batch size since this setup was more suitable with the sample amount of dataset we used. Various numbers of epochs were experimented with to obtain the best performance results. They tried to increase the number of epochs to 100; however, it took more running time without significant progress in accuracy.	Convolution layers, pooling layers, flattening, and multilayer perceptrons made up the majority of the CNN design. CNNs used fully connected neural networks to classify the input photos after automatically extracting features from them. The convolution and pooling layers were used to extract features. Following the application of filters to these layers, the image’s features were acquired, and the classification phase began.	[19]
Huang et al.—2020	Deep Learning (CNN) They employed transfer learning technology. With this method knowledge from other tasks is transferred to the current task, requiring less data for learning and adaptation to the target task.	GoogLeNet ResNet DenseNet	[20]
Zhang et al.—2022	Deep Learning The UNet22 design offered a precise location for picture semantic segmentation in medical image segmentation. High segmentation and classification accuracy were achieved by the deformable convolution layer, which implemented the free-form deformation of the feature learning process. UNet introduced skip connections between the encoder feature maps and the decoder feature maps at the same scale in contrast to the standard encoder–decoder segmentation models.	The multiclass bone marrow cell segmentation performance of CMLcGAN was satisfactory. Following segmentation, five statistical features were chosen, and a conventional threefold cross-validation with 100 repetitions was carried out.	[22]
Dese et al.—2021	k-means clustering Marker-controlled watershed segmentation Morphological operations	SVM classifies the provided inputs using optimum hyperplanes. Together with the class descriptors, the hyper plane—also known as support vectors—is constructed from the training instances. Like a line dividing a 2D plane into two sections, these hyper planes separate the positive samples from the negative samples.	[23]

**Table 2:** AI’s application in CML management.

Morphometric Analysis			
	Material	AI	Ref.
	Peripheral blood	CNN	[18]
	Blood	CNN	[19]
	Bone marrow blood	CNN	[20]
	Bone marrow biopsy	cGAN	[22,23]
Immunophenotyping			
	Periferal and bone marrow blood	ML	[26]
	Bone marrow blood	SVM	[27]
Karyotyping			
	Bone marrow blood	Specific algorithm	[28]
Integrated data			
	Aim		
	Prognosis	ML	[30]
	Prediction	ANN	[31]
	Prediction	ML	[32]
	Response to treatment	LEAP	[33]
	Response to treatment	SVM	[34]
	Response to treatment	RF	[35]

CNN: convolutional neural network; cGAN: conditional generative adversarial network; ML: machine learning; SVM: support vector machine; ANN: artificial neural network; LEAP: leukemia artificial intelligence program; RF: random forest.

**Table 3:** Use of AI in the study of toxicity and side effects of TKis, management of drug resistance and generation of new drugs.

Toxicity and Side Effects			
Target	Drug	AI	Ref.
Gamma-Tocotrienol	Asciminib	Deep learning	[42]
Drug resistance			
Aim			
Resistance profiles against 8 drugs	Axitinib, Bosutinib, Dasatinib, Erlotinib, Gefitinib, Imatinib, Nilotinib, and Ponatinib	Machine learning	[37]
Overcome T315I resistance	Imatinib mesylate, nilotinib, dasatinib	Machine learning	[38]
Generation of new drugs			
Evaluation of bioactivity scores		Support Vector Machine	[51]
Potential binding sites		Support Vector Machine Cavity Plus	[52]
Allosteric drug development		FTMap	[53]
Identification of binding hot spot		FTMap	[54]
Evaluation of allosteric and non-allosteric inhibitors		Random forest, Support Vector Machine, Deep neural network	[55]
Production of TKIs inhibiting TKI-resistant cells		Machine learning	[57]
Evaluation of Withaferin A as TKi		Algorithm of Glide, Visual Molecular Dynamics	[60]

## Data Availability

Not applicable.
